# Natural Holobiome Engineering by Using Native Extreme Microbiome to Counteract the Climate Change Effects

**DOI:** 10.3389/fbioe.2020.00568

**Published:** 2020-06-04

**Authors:** Rodrigo Rodriguez, Paola Durán

**Affiliations:** ^1^Biocontrol Research Laboratory, Universidad de La Frontera, Temuco, Chile; ^2^Scientific and Technological Bioresource Nucleus, Universidad de La Frontera, Temuco, Chile

**Keywords:** microbiome, climate change, microbiome engineering, sustainable agriculture, microbiome transferring, crop productivity

## Abstract

In the current scenario of climate change, the future of agriculture is uncertain. Climate change and climate-related disasters have a direct impact on biotic and abiotic factors that govern agroecosystems compromising the global food security. In the last decade, the advances in high throughput sequencing techniques have significantly improved our understanding about the composition, function and dynamics of plant microbiome. However, despite the microbiome have been proposed as a new platform for the next green revolution, our knowledge about the mechanisms that govern microbe-microbe and microbe-plant interactions are incipient. Currently, the adaptation of plants to environmental changes not only suggests that the plants can adapt or migrate, but also can interact with their surrounding microbial communities to alleviate different stresses by natural microbiome selection of specialized strains, phenomenon recently called “*Cry for Help*”. From this way, plants have been co-evolved with their microbiota adapting to local environmental conditions to ensuring the survival of the entire holobiome to improve plant fitness. Thus, the strong selective pressure of native extreme microbiomes could represent a remarkable microbial niche of plant stress-amelioration to counteract the negative effect of climate change in food crops. Currently, the microbiome engineering has recently emerged as an alternative to modify and promote positive interactions between microorganisms and plants to improve plant fitness. In the present review, we discuss the possible use of extreme microbiome to alleviate different stresses in crop plants under the current scenario of climate change.

## Introduction

According to the Organization for Economic Cooperation and Development (OECD), agriculture is considered one of the most essential economic, social and environmental activities for human beings. Until now, agricultural and livestock products has been able to respond to the growing world demand. However, it is estimated that by 2050 the world population would reach 9.7 billion people and that crop productivity should increase approximately 60–100% to meet this demand of food (Hunter et al., 2017; United Nations, 2019). This is due to climate change and climate-related disasters have a direct impact on biotic and abiotic factors that govern agroecosystems, compromising the global food security (Challinor et al., 2014; FAO, 2016a; Dresselhaus and Hückelhoven, 2018; Cavicchioli et al., 2019; Raza et al., 2019). For this reason, the new green revolution is required to achieve future food security, where new concepts and approaches are needed to achieve a more sustainable development of agriculture.

To date, many studies have investigated biotechnological solutions to counteract the negative effect on crop yield. Since the last 20 years, isolation through culture-dependent techniques and inoculation of cultivable Plant Growth-Promoting Microorganisms (PGPM) have substantially improved plant fitness under laboratory conditions (Adesemoye et al., 2008; Barra et al., 2016, 2019; Viscardi et al., 2016). However, the low extraction efficiency of the culture-dependent methods (<1%) and the limited establishment of the microorganisms under field conditions, represent an important limitation for their massive use (Alabouvette et al., 2009; Toju et al., 2018b). Nowadays, the advances in meta-omics have significantly improved our understanding about the composition, function and dynamics of cultivable and non-cultivable microorganisms in agroecosystems (Esposito et al., 2016; Meena et al., 2017; Wang et al., 2017; Toju et al., 2018a), allowing a better understanding of the microbiomes associated in different ecosystems, the participation of each member of these complex microbial communities and the role of keystone microbial taxa. However, the mechanisms that govern microbe-microbe and microbe-plant interactions are incipient and few studies have used this ability to transfer microbiomes (e.g., performing soil transplants) to improve crop production. In contrast, in human health the manipulation and transplants of microbiomes or core microbiomes is currently applied to solve diseases (Borody and Khoruts, 2012; Kelly et al., 2016; Paramsothy et al., 2017; Lev-Sagie et al., 2019).

Our current knowledge about adaptation of plants to environmental changes not only suggests that plants can adapt or migrate, but can also interact, modify and select specific microbial communities to mitigate the negative effect of different stresses, phenomenon recently called “*Cry for Help*” (Bakker et al., 2018; Berendsen et al., 2018; Carrión et al., 2019; Li X. et al., 2019; Rolfe et al., 2019). Therefore, the modification of these interactions between microorganisms and plants could represent a promising alternative to mitigate the negative effects of climate change on food crops. In this context, microbiome engineering has recently emerged as an alternative to promote positive interactions between microorganisms and host plants (Mueller and Sachs, 2015; Sheth et al., 2016; Foo et al., 2017; Timmusk et al., 2017; Trivedi et al., 2017; Hussain, 2019). Recently, Jochum et al. (2019b) have performed host-mediated microbiome engineering to attribute drought resistance in wheat plants, which demonstrates the potential for modification of native microbiomes to attribute crop resistance. Thus, the transfer of whole microbiomes to improve crop yields under different stress situations could represent an unparalleled advance to start a new revolution in the bioinoculants development (Santoyo et al., 2017; Woo and Pepe, 2018). In this context, microbiomes of extreme environments could represent an unequaled source of stress-ameliorating due to the strong selective pressure suffered by microorganisms in extreme environments (Durán et al., 2019). In this review, we highlighted the main characteristics of natural microbial engineering focalizing in extreme microbiomes to improve crop resistance to different stresses in the current scenario of climate change events.

## Climate Change and Crop Productivity: Impacts and Priority Research

Climate change and catastrophic events have negatively influenced the plant physiology (Challinor et al., 2014; Wang et al., 2018; Raza et al., 2019), generating important economic losses in the agri-food sector in the last decade (FAO, 2016a; Huong et al., 2018). In this context, drought stress is the most worrisome stress in the current scenario of climate change (FAO, 2016b). Studies conducted with heat and drought stresses (combined stresses) on *Solanum lycopersicum*, showed that drought stress has a predominant negative effect on plant survival (Zhou et al., 2017, 2019). For example, at morpho-physiological level, it is known that plants decrease leaf area and stem length, reduce leaf water potential, decrease stomatal conductance, decrease net photosynthetic rate and loss of turgor and osmotic adjustment as a consequence of lack of water with a concomitant reduction of plant productivity (Jangid and Dwivedi, 2016; Hussain et al., 2018; Kumar et al., 2018). From a biochemical point of view, plants are able to increase the activity of antioxidant enzymes (Superoxide dismutase, SOD; Catalase, CAT; and Peroxidase, POD), temporarily reduce photochemical efficiency, reduce the accumulation of reactive oxygen species (ROS), decrease Rubisco efficiency and increase the accumulation of Proline, Polyamine and Trealose (Ammar et al., 2015; Kumar et al., 2018). At molecular level, plants can increase the expression of the biosynthetic genes of the phytohormone abscisic acid (ABA), synthesize specific proteins such as dehydrins and late embryogenesis abundant (LEA) proteins and increase the expression of transcription factors such as *DREB*, *WRKY*, and *NAC* that produce resistance against drought stress (Liu G. et al., 2014; Liu Y. et al., 2014; Sun et al., 2015; Yang et al., 2017; Kumar et al., 2018). To date, ABA is perhaps the most important phytohormone in the regulation of water use by plants under drought conditions. It is currently known that ABA is a key intermediary for the control of stomatal closure under water scarcity conditions (Mishra et al., 2006; Mega et al., 2019) and is also a signaling intermediary that induces the expression of genes such as *RD22*, *RD29A*, *RD29B*, *KIN2*, *RAB18*, and *PYL8* that play an important role in response and tolerance against dehydration (Abe et al., 1997; Ramírez et al., 2009; Cutler et al., 2010; Yao et al., 2012; Lim et al., 2013). In the last decades, interaction between UVs (UV-B) exposition and drought stress in plants has been evidenced. For example, Alexieva et al. (2001) showed that despite UV-B radiation has stronger stress effectors than drought. However, both acted synergistically in order to induce protective mechanisms to reduce the stress in wheat and pea plants. In contrast, Novotná et al. (2016), shown that drought led to a significant reduction of above-ground biomass, particularly under ambient UV radiation. Thus, the combined effect of drought and UV radiation which may result in an enhance or vice-versa alleviation of drought impact. This is important to consider due to Bais et al. (2015) estimated that by the end of the 21st century the most populated areas of the northern hemisphere could increase 10–20% in UV radiation. Sunlight provides the energy necessary for plant growth through photosynthesis, however, high energy light and, in particular, UV-A (315–400 nm) and UV-B (280–315 nm) can produce damage to plants through structural modifications in DNA, protein denaturation, damage to membranous organelles (chloroplasts, mitochondria and nucleus) and cause oxidative stress (Müller-Xing et al., 2014; Sharma et al., 2017; Tossi et al., 2019). UV-B has been considered as the most harmful type of radiation for plants, morphological changes have been observed as thicker leaves, shorter petioles and lower chlorophyll content (Zhu et al., 2010; Inostroza-Blancheteau et al., 2014; Robson et al., 2015). Studies conducted in recent years show that high UV-B irradiation can even decrease CO_2_ assimilation, decrease photochemical efficiency of photosystem II (PSII), reduce electron transport rate (ETR) and limit the productivity of some crops (Basahi et al., 2014; Wang et al., 2015; González-Villagra et al., 2020). Our current knowledge has determined that plants can counteract the negative effect of UV-B through a series of morphological and molecular changes, such as, the biosynthesis of phenolic acids and flavonoids induced by a UV-B photoreceptor called UV RESISTANCE LOCUS 8 or UVR8 (Coffey et al., 2017; Yang et al., 2018; Kondou et al., 2019; Tossi et al., 2019). Nowadays, different research groups have proposed novel biotechnological methods to mitigate the effect of abiotic stress on agricultural crops, among them the use of PGPM are widely studied (Calvo-Polanco et al., 2016; Vurukonda et al., 2016; Ullah et al., 2017; Etesami and Maheshwari, 2018; Bernardo et al., 2019; Bahadur et al., 2019; Mathur et al., 2019; Mickan et al., 2019). Several studies related with PGPM isolated from extreme environment has been widely reported (Table 1), such is the case cold desert such as Antarctic, arid desert, volcanoes and hydrothermal environments. For example, Timmusk et al. (2014) have used *Bacillus thuringiensis* AZP2 and *Paenibacillus polymyxa* B isolated from hostile environments to induce drought resistance to wheat crops. Moreover, Lim and Kim (2013) have used *Bacillus licheniformis* K11 to induce drought resistance through the action of Auxins and ACC deaminase produced by this strain. Furthermore, Khan et al. (2018) used PGPM isolated from the rhizosphere of rainfed area (Karak) in Pakistan (*Bacillus cereus* and *Planomicrobium chinense*) combined with salycilic acid to improve *Helianthus annuus* resistance. Recently, Jochum et al. (2019a) isolated and characterized two bacterial strains (*Bacillus* sp. and *Enterobacter* sp.) with a high potential to lag the effects of drought on wheat (*Triticum aestivum*) and corn (*Zea mays*) seedlings. In the case of arbuscular mycorrhizal fungi (AMF), for example, Meddich et al. (2015) used different mycorrhizal fungi to improve the performance of *Phoenix dactylifera* against water deficit, finding a significant increase in peroxidase and polyphenolxidases in the roots of mycorrhized palms enhancing the survival of plant in these stress conditions. In addition, Li J. et al. (2019) have used symbiote fungi to confer resistance to C3 grass *Leymus chinensis* and in C4 grass *Hemarthria altissima* under water deficit condition. Our current knowledge about the role of AMFs to help plants withstand drought stress is mainly involved in direct absorption of water by hyphae and their transfer to the host plant, increasing water content and eliminating the generation of ROS and the production of biomolecules and enzymes with antioxidant capacity (Huang et al., 2017; Liu C. Y. et al., 2017; Liu Y. et al., 2017; Bahadur et al., 2019). In the case of biotechnological tools to mitigate the damage caused by UVs radiation, only preliminary studies have been highlighting the important role of phyllosphere microorganisms, which has been co-evolved on the surface of plant leaves, developing defense mechanisms to counteract the harmful effects of UV-B. For example, it has been shown that *Clavibacter michiganensis* present on the surface of *Arachis hypogaea* leaves are highly resistant to UV-B radiation due to carotenoid production and the expression of CAT and SOD (Jacobs and Sundin, 2001; Jacobs et al., 2005). Additionally, *Enterobacter cloacae* isolated from the rice surface also has a great capacity to resist UV-B radiation due to the expression of certain proteins (Kumar et al., 2016). Despite biostimulant-based technologies are promising to improve crop yields (Melusá et al., 2012; Pratap et al., 2016), the use of PGPM is largely limited by poor success in field conditions. This limitation is mainly due to the abundance and functional diversity of native soil microorganisms, allowing them to occupy most of the available ecological niches, so that attempts to introduce new microorganisms are limited (Felici et al., 2008; Alabouvette et al., 2009; Martinez-Viveros et al., 2010; Trabelsi et al., 2011; Lindemann et al., 2016; Toju et al., 2018b). The niche overlap between an inoculant and resident microorganisms seems to be limited even with resident microorganisms that are phylogenetically related to the inoculant (Castro-Sowinski et al., 2007). However, some studies showed that inoculation with microbial consortia is a more effective approach than inoculation with a single strain, since microorganisms seem to function synergistically and are able to compete for certain ecological niches (Berendsen et al., 2018; Woo and Pepe, 2018). For this reason, the use of complete microbiomes or core microbiomes (both cultivable and non-cultivable strains) could represent a promising alternative to modify the microbial communities native to agroecosystems in order to counteract the negative effects of climate change.

**TABLE 1:** Plant Growth Promoting Microorganisms isolated from extreme environment to improve crop production.

Source	Strains	Application	Reference
Chilean hydrothermal	*Bacillus* sp. *Geobacillus* sp.	Phytases production	Jorquera et al., 2018
Arid soil of Saudi Arabia	*Pseudomonas stutzeri Bacillus* sp. *Bacillus subtilis Enterobacter* sp. *Enterobacter cloacae Pseudomonas putida Bacillus subtilis* subsp. *inaquosorum Enterobacter* sp.	Pathogen inhibition	El-Sayed et al., 2014
Qinghai-Tibetan plateau	*Bacillus* sp. *Bacillus velezensis*	Improves plant growth under cold stress	Zubair et al., 2019
Salty soil, Utah	*Halomonas* sp. *Bacillus* sp.	Improves plant growth under saline stress	Kearl et al., 2019
Extreme saline-alkali soil of Qinghai Province	*Bacillus amyloliquefaciens*	Pathogen inhibition Nitrogen fixation and IAA production	Wu et al., 2019
Antarctic plants	Symbiont consortium of *Arthrobacter* sp., *Planoccocus* sp., *Penicillium chrysogenum* and *P. brevicompactum*	Improves plant growth	Acuña-Rodríguez et al., 2019
Semiarid regions in the northeast of China	*Pseudomonas fluorescens Enterobacter hormaechei Pseudomonas migulae*	Seeds germination and seedling growth	Niu et al., 2018
Rhizosphere of *Hordeum maritimum* growing in saline soil	*Bacillus mojavensis Bacillus pumilus Pseudomonas fluorescens*	Improves plant growth	Mahmoud et al., 2017
Volcano soils	*Ochrobactrum* sp.	Improves plant growth	Mishra et al., 2017
Hypersaline soils in Tunisia	*Halomonas* sp.	Improves plant growth	Mapelli et al., 2013
Cold Desert of north western Indian Himalayas	*Bacillus licheniformis Bacillus muralis Desemzia incerta Paenibacillus tylopili Sporosarcina globispora*	Improves plant growth	Yadav et al., 2016
Rhizosphere of halophytes (*Halimione portulacoides* and *Salicornia ramosissima*)	*Vibrio spartinae Marinobacter sediminum Vibrio parahaemolyticus Vibrio neocaledonicus Thalassospira australica Pseudarthrobacter oxydans*	Improve seed germination	Mesa-Marín et al., 2019
Salt-accumulating halophyte *Salicornia* sp.	*Staphylococcus* sp.	Improves plant growth	Komaresofla et al., 2019
Hypersaline environment, Sambhar lake, India	*Firmicutes Proteobacteria Actinobacteria*	Phosphate solubilization and production of siderophore, ammonia, hydrogen cyanide, ACC-deaminase and indolic compounds	Sahay et al., 2012
Saline desert of Kutch, India	*Bacillus licheniformis*	Improves plant growth	Goswami et al., 2014
Antarctic Soil	*Pseudomonas* sp.	Phosphatase production	Berríos et al., 2012
Roots of Antarctic plants	*Tapesia* sp. *Mollisia* sp. *Incertae sedis Oculimacula yallundae*	Increase availability of nitrogen	Upson et al., 2009
Fungi from Antarctic plants	*Penicillium chrysogenum Penicillium brevicompactum*	Improves *Lactuca sativa* L. growth	Molina-Montenegro et al., 2016

## Natural Microbiome Engineering. the New Horizons to Alleviate Climate Change Consequences

Currently, several studies have been highlighted the important role of root endosphere, which are able to recruit desirable microorganism from soil to improve plant fitness and yields of crops (Kasotia and Choudhary, 2014; Molina-Montenegro et al., 2016; Berg and Koskella, 2018; Durán et al., 2018; Lata et al., 2018; Orozco-Mosqueda et al., 2018; Carrión et al., 2019; Dubey and Sharma, 2019; Harman and Uphoff, 2019). To date, some studies have identified the structure and function of the microbiome in agricultural crops including barley (Bulgarelli et al., 2015), soybean (Mendes et al., 2014; Rascovan et al., 2016), maize (Aira et al., 2010; Gomes et al., 2018), wheat (Donn et al., 2015; Chen et al., 2018) and rice (Edwards et al., 2015). In fact, studies showed that plants can select their microbiome and desirable traits can be transmitted. For example, multigenerational experimental systems with *Arabidopsis thaliana* have been used to select soil microbiomes that induce different flowering times. Microbiome selected from the 10th generation of *A. thaliana* was inoculated in *A. thaliana* and *Brassica rapa* soils and the characteristics were transferred (Panke-Buisse et al., 2015). Thus, plant microbiome is gaining considerable interest since they play an important role in the regulation of plant metabolism (Pieterse et al., 2014; Mueller et al., 2016), where plants can adjust their microbiome and specifically recruit the microorganisms involved in plant fitness with a subsequent assembly of protective specific microbiota, a phenomenon that was recently called “*Cry for Help*” (Bakker et al., 2018; Berendsen et al., 2018; Li X. et al., 2019; Rolfe et al., 2019). Although this new theory is mainly related to biotic stresses, Liu et al. (2015) showed that the respiratory metabolic capacity of mycorrhizal rice (*Oryza sativa*) under low-temperature stress accelerated the biosynthesis of strigolactone to recruit AMF (*Glomus intraradices*). On the other hand, in a study published by Jochum et al. (2019b), it was possible to modify the phenotype of *Triticum aestivum* subsp. *aestivum*, using host-mediated microbiome engineering as a strategy to improve the resistance of this crop to drought stress. therefore, this phenomenon could eventually be associated with different abiotic stress such as high radiation, freezing, among others.

**FIGURE 1:**
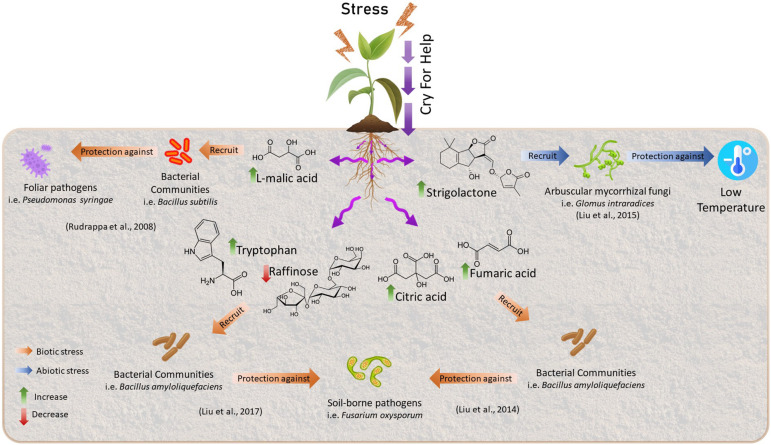
Examples of Cry for help process. Plants undergoing environmental stress could change their radical exudation profiles of primary and secondary metabolites to recruit beneficial microorganisms to counteract the negative effect of the stress.

The modification of the microbial communities is given by the alteration of the radical exudation profiles of primary and secondary metabolites with biocidal and/or semiochemical activities, which influences the microbiome recruiting specific microorganisms and their microbial activities (Rudrappa et al., 2008; Hassan and Mathesius, 2012; Massalha et al., 2017; Rolfe et al., 2019). This restructuring of microbiome involves the exudation of compounds that could serve as a substrate for microbial growth, elicit chemotactic responses and facilitate root colonization or inhibit the growth of some microbial groups through the release of antimicrobial compounds (Figure 1). Furthermore, exudates can interact with microbial quorum sensing systems causing the release of metabolites derived from microbial metabolism (Rolfe et al., 2019). To date, the secondary metabolites from Shikimate biosynthetic pathway and derivatives of Isopentenodiphosphate (IPP) pathway have been most reported (Rolfe et al., 2019). The concept of cry for help is based on many studies, for example, it has been shown that Arabidopsis plants infected with the foliar pathogen, *Pseudomonas syringae*, increase the secretion of L-malic acid toward the rhizosphere, selectively recruiting the rhizobacterium *Bacillus subtilis* promoting the formation of biofilms in the roots and consequently increasing plant defenses (Rudrappa et al., 2008). Some years after, studies conducted by Lakshmanan et al. (2012) demonstrated that foliar infection by *Pseudomonas syringae* induced the expression of aluminum-activated malate transporter 1 (*ALMT1*) that increases the expression of L-malic acid toward the rhizosphere which caused a systemic resistance response induced in plants against pathogen by recruiting of *B. subtilis*. In addition, a study by Berendsen et al. (2018) shows that *A. thaliana* recruit three bacterial phylum (Proteobacteria, Firmicutes, and Bacteroidetes) in the rhizosphere after activation of foliar defense by the downy mildew pathogen, *Hyaloperonospora arabidopsidis*.

Thus, currently it is well known that the survival of plants depends on the interaction with the holobiont. However, the protective function is not carried out by all microorganisms, but rather by some microbial groups that, due to additive or synergistic effects, are fundamental to the functioning of this protection network. These key microorganisms within the microbiome, called “core microbiome”, forms strong facultative and mutualistic interactions with the other microorganisms inside the microbiome to generate the protective effect. The core microbiome concept was introduced in human microbiome studies to define a relatively stable community that contributes to important biological functions (Human Microbiome Project Consortium, 2012). The core microbiome are small fractions of OTUs (operational taxonomic units) that represent <∼20% of the total microbial diversity but that represents more than ∼90% of the relative abundance of bacterial and fungal OTUs assembled in the different organs of the plant, which maintain their high relative abundance during the development of the plant (Lundberg et al., 2012; Armanhi et al., 2016; de Souza et al., 2016; Armanhi et al., 2018; Toju et al., 2018a, b). The importance of the core microbiome has been tested experimentally in recent years, for example, Niu et al. (2017) evaluated the role of a simplified synthetic microbial consortium formed by seven strains of four phyla (*E. cloacae*, *Stenotrophomonas maltophilia*, *Ochrobactrum pituitosum*, *Herbaspirillum frisingense*, *Pseudomonas putida*, *Curtobacterium pusillum*, and *Chryseobacterium indologenes*) identified by culture-dependent techniques. The authors showed that elimination of *E. cloacae* led to the complete loss of the community, suggesting an important role of the key species in the functioning of the total community. Studies conducted by de Souza et al., 2016 found that a core microbiome composed of 25% of the total microbial diversity of endophytic microorganisms (bacteria and fungi) and 15% of exophytic microorganisms, represented more than 90% of the relative abundance of bacteria and fungi found in the different organs of the sugarcane plant. Therefore, identify, add or modify the core microbiome in different agriculture crops represents a promising alternative to counteract the negative effects of climate change. In fact, strengthening the core microbiome is defined among the five research priorities “For harnessing plant microbiomes in sustainable agriculture” (Busby et al., 2017). From this point of view, different studies showed that the synthetic design and transplantation of core microbiomes can improve plant fitness. For example, Armanhi et al. (2018) identified and isolated the core microbiome from roots and stems of sugarcane and then was inoculated into maize plants. As a result, members of the synthetic community efficiently colonized plants organs, displacing the natural microbiota and dominate at 53.9% of the rhizosphere microbial abundance generating a 3.4-fold increase in plant biomass compared to non-inoculated plants. In natural environment, plants have evolved together with their microbiota adapting to local environmental conditions ensuring the survival of the holobiont (Cordovez et al., 2019). However, few studies have attempted to identify these processes in plants of extreme environments. The native plants microbiome of extreme environments could represent an unequaled source of stress-ameliorating microorganism and the natural microbiome engineering by using extreme microbiome could represent a promising and eco-friendly alternative to ensure the global food security.

## Extreme Microbiome to the Service of Sustainable Agriculture

The plant holobiome has been developed over the centuries to adapt to the different terrestrial biomes. Cold environments, such as Antarctic, and dry environments, such as Deserts, have aroused great curiosity regarding the assembly of microbial communities and microbe-plant interactions. Although the study of the microbiome in extreme environments is still an incipient area, some authors have begun to identify the complex interactions between the microbiome and vegetation associated with these hostile environments. For example, desert (also known as dry/arid environments) represent about a third of the planet’s biomes, which are characterized by significant absence of rainfall, extreme fluctuations in temperature, generally low nutrient status, high levels of incident UV radiation and strong winds (Chamizo et al., 2012; Stomeo et al., 2013). The microorganisms associated with these environments are generally represented by Bacteria, Fungi, and Archaea (Makhalanyane et al., 2015; Fernández-Martínez et al., 2019). Bacterial communities present in desert soils typically contain a number of ubiquitous phyla including Actinobacteria, Bacteroidetes and Proteobacteria (Fierer et al., 2009). In the case of fungi, most of the studies have identified phyla that included members of Basidiomycota and Ascomycota, with high taxonomic diversity, both thermophilic and thermotolerant fungi (Fierer et al., 2012; Makhalanyane et al., 2015). Archeal taxa are relatively rare across many environments but seem to be particularly abundant in desert soils, with the Thaumarchaeota phylum the most representative (Fierer et al., 2012; Marusenko et al., 2013). Although we know little about the microbial diversity of desert environments, new metagenomic data have shown functional diversity and a large abundance of genes involved in biogeochemical cycles that, although much less than other terrestrial biomes, could generate functional trophic chains (Makhalanyane et al., 2015).

Some authors have investigated the use of PGPM from these environments, for example, El-Sayed et al., 2014, isolated native bacteria from rhizospheric arid soils and evaluated both growth-promoting capabilities and antagonistic potential against fungi and phytopathogenic nematodes. They found bacteria that exhibited capacities to fix atmospheric nitrogen, produce ammonia, indole-3-acetic acid (IAA), siderophores, solubilize phosphate and zinc, and showed a potential antagonist against some phytopathogenic fungi and a species of nematodes (*Meloidogyne incognita*) to varying degrees. Moreover, Niu et al., 2017 identified bacterial strains of foxtail millet (*Setaria italica* L.), a drought-tolerant crop grown in semi-arid regions in northeast China. They observed that four isolated strains had the ability to generate ACC deaminase, as well as tolerance to drought. In the case of microbiome, some plant-associated microbiomes have been identified in deserts habitats, for example, Coleman-Derr et al. (2016) identified microbiomes of three *Agave* species (*Agave tequilana* FAC Weber, *Salmian Agave* Otto ex Salm subsp. *crassispina* (Trel.) Gentry, and *Agave deserti* Engelm) distributed in central Mexico and in southern California, finding microorganisms capable of conferring resistance to high temperatures and low water availability. Moreover, Fonseca-García et al. (2016) identified the holobioma of succulent plants (Family *Cactaceae*) native to arid and semi-arid ecosystems which also are represent microorganisms capable of conferring drought resistance.

Other desert environments such as the Atacama Desert in Chile, have aroused great scientific interest in recent years. This desert, considered the driest in the world, has a great microbial diversity that is still largely unknown taxonomically (Bull et al., 2016). Recently, some studies have investigated in the microbiome dynamics associated with the “Atacama Flowering Events,” which corresponds to an explosive bloom of dormant desert plants produced by the presence of water as precipitation (Vidiella et al., 1999). Studying this phenomenon, it was discovered that some bacterial groups and their activity can influence the growth and flowering of native plants (Araya et al., 2020; Astorga-Eló et al., 2020). Similarly, cold desert such as Antarctic have also been studied. The Antarctic pristine environment is the most extreme land on the planet and represents an interesting and unique habitat for the colonization and survival of microbial life. The first expeditions and studies in Antarctica suggested that this territory as sterile and with limited microbial activity (Cameron et al., 1968). However, in the recent years a large number of studies have demonstrated a high diversity of microorganisms with structured trophic chains that form functional microbial communities (Cary et al., 2010; Teixeira et al., 2013; Niederberger et al., 2015, 2019; Jorquera et al., 2016; Pudasaini et al., 2017; Durán et al., 2019). Over the years, a number of particular characteristics of Antarctic environment have been discussed and investigated. The combination of an extensive glacial layer, intense katabatic winds, high radiation and extremely low precipitation rates (low 2%) makes them the oldest, cold, dry and hostile territory for microorganisms. This environment is dominated by strong gradients in temperature, salinity (35–150%), and irradiation (<0.1% to 1–5% UV radiation), properties highly variable and ultimately governed by air temperature and snow cover. This strong selective pressure leads to the evolution of novel mechanisms for stress tolerance by indigenous microorganisms, forming an important ecological niche. The extreme environmental conditions of Antarctica greatly limit the establishment of plants. However, the Maritime Antarctic region (mainly in the Antarctic Peninsula) provides favorable weather conditions for the establishment of the only two vascular plants: *Deschampsia antarctica* and *Colobanthus quitensis* (Alberdi et al., 2002).

To date, some studies have identified the role that extreme microorganisms play in the nutrition and survival of these vascular plants. For example, Antarctic *Pseudomonas* were characterized that help solubilize phosphate sources to enhance phosphorus absorption by *D. antarctica* (Berríos et al., 2012; Yarzábal et al., 2018). In the case of fungi, studies have mainly focused on endophytic fungi. For example, fungi with dematiaceous septate hyphae (Dark Septate Endophytes) capable of mineralizing peptides and amino acids have been found in the rhizosphere of *D. antarctica*, indicating that they increase the availability of nitrogen for the plant (Upson et al., 2009). Moreover, endophytic symbiont yeasts (*Cryptococcus victoriae*, *Cystobasidium laryngis*, *Rhodotorula mucilaginosa*, *Sporidiobolus ruineniae*, and *Leucosporidium aff. golubevii*) have been identified in leaves of both vascular plants that could directly or indirectly promote the fitness of host plants (Santiago et al., 2017). Recently, studies by Ramos et al. (2018) demonstrate that endophytic fungi present in *C. quitensis* modulate the content of salicylic acid, jasmonate, indole-3-acetate and ABA in shoot tissue of plants exposed to UV-B radiation, which would indicate that these endophytic fungi could modulate the hormonal content of *C. quitensis* to improve its ecophysiological performance under high UV-B radiation. Thus, the plant-associated microorganisms can play a crucial role to ensure the plant survival (Upson et al., 2009; Torres-Díaz et al., 2016; Gallardo-Cerda et al., 2018; Ramos et al., 2018; Ballesteros et al., 2020).

Nowadays, the use of Antarctic microorganisms to enhance the yield of agricultural crops under this new scenario of climate change is more frequent. In the case of Emerging Infectious Disease, Melo et al. (2016) isolated epiphytic bacteria from *D. antarctica* that inhibit *Botrytis cinerea*, and Javeria et al. (2014) showed that lichen forming fungi isolated from *Everniastrum cirrhatum* lichen have important antimicrobial properties against *Fusarium moniliforme*, *Fusarium oxysporum* and *Fusarium udum*. On the other hand, Molina-Montenegro et al. (2016), show the potential of some Antarctic endophytic fungi isolated from *C. quitensis* and *D. antarctica* to improve the net photosynthetic rate and water absorption under drought conditions in cultivars of *Lactuca sativa* L. var. *Longifolia*. In addition, a recent study conducted by Acuña-Rodríguez et al. (2019) formulated a consortium of microorganisms formed by two growth-promoting rhizobacteria of the genus *Arthrobacter* and *Planoccocus* and two endophytic root fungi *Penicillium chrysogenum* and *Penicillium brevicompactum* that worked effectively to reduce saline stress in pepper, lettuce, onion and tomato plants. The results of these experiments demonstrate a high capacity to enhance crop yield under biotic and abiotic stresses by some microorganisms. However, as discussed earlier, the use of few microorganisms generally represents low success in the field due to the low adaptability and competition of introduced microorganisms with the native microorganisms.

**FIGURE 2:**
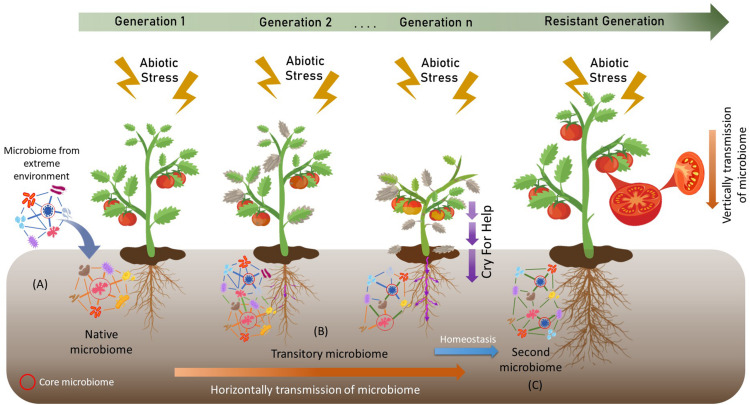
Artificial selection of microbiomes by host-mediated and multigenerational selection. **(A)** The incorporation of an extreme microbiome modifies the native microbiome. **(B)** This new transitory microbiome is transmitted horizontally through the soil and undergoes alterations through the processes of Cry For Help. **(C)** After time, the microbiome reach homeostasis forming a second microbiome that helps to alleviate the negative effects of climate change.

A recent study by Molina-Montenegro et al. (2019) suggests that *D. antarctica* and *C. quitensis* can modify their rhizosphere microbiome under different stress conditions. Similarly, Citlali et al. (2018) suggest that the rhizosphere and phyllosphere of CAM plants differentially benefit their host plants to succeed in drylands. The transfer of microbiomes or core microbiomes could mitigate the negative effects of different biotic and abiotic stresses on agricultural crops. However, as far as we know, the use of microbiomes or core microbiomes from this hostile environment to improve the resistance of agricultural crops has not been investigated. Different studies have shown that it is possible to transfer complete microbiomes by transplants of small portions of soil (Lau and Lennon, 2012; Panke-Buisse et al., 2015; Tkacz et al., 2015; Yergeau et al., 2015; Calderón et al., 2017; Howard et al., 2017). For example, Howard et al. (2017) demonstrated a successful transfer of soil microbiomes from an urban forest in Ithaca, NY, United States. After 3 weeks of incubation, the pots with 5% v/v soil presented a similar composition of microbial communities to the transferred soil. In addition, as we have already discussed, microbiomes are capable of modifying the metabolism and phenological stages of some plants. Therefore, the combination of extreme microbiomes, together with processes such as cry for help, where the plant recruits the microorganisms it needs to resist stresses, could generate new and naturally selected entities that help crops withstand these unfavorable conditions (Figure 2). The identification of these new central entities through the new “meta-omics” techniques could enhance knowledge about the dynamics of microbial communities in specific situations in order to perform more precise and more resilient bioinoculants in the soil.

## Impact of the Application of Microbiomes in Soils

In the soil, the microbial communities are responsible for nutrient cycling, absorption and release of nutrients, mineralization and processing of organic and inorganic compounds (Madsen, 2011; Falkowski et al., 2012; Jansson and Hofmockel, 2019). These processes are highly dynamic and depend largely on environmental conditions, but also on the interactions between the biotic components of the system (Churchland and Grayston, 2014). The microbe-microbe and microbe-plant interaction is essential for the proper functioning of the biogeochemical cycles. Some studies indicate that the introduction of microorganisms can positively influence native microbial communities and soils processes (Miethling et al., 2000; Johansen and Binnerup, 2006; Singh et al., 2010; Castro-Sowinski et al., 2007; Bargaz et al., 2018). For example, the inoculation of with *Azospirillum* sp. improves the efficiency of nitrogen absorption in soil, influencing the metabolic activity of other microbial communities (Salamone et al., 2010, 2012). Besides, the inoculation of *Azospirillum* sp. and *Pseudomonas* sp. can increase in the diversity of microbial communities in wheat crops, which produces large changes in profiles of carbon-source utilization modifying the soil carbon pools, generating labile organic matter and increasing soil fertility (Naiman et al., 2009).

In the case of complete microbiomes, the information is limited and even more in terms of agroecosystems. Some studies have begun to analyze the effect of microbiome transferring in different scenarios. For example, Zhao et al. (2014) used soil transplants, and consequently their microbiomes, in order to study changes in soil biogeochemical cycles. They showed that after 4 years of soil transplantation, microbial functional diversity and the processes involved in the nitrogen cycle increased considerably. Surprisingly, genes associated with the nitrogen and carbon processing increased in abundance, coinciding with a greater potential for soil nitrification and carbon sequestration. On the other hand, Yergeau et al. (2015) showed that the microbiome transferring helps the growth of willows on petroleum-contaminated soils. However, after 100 days of incorporation of the microbiome, the microbial communities tend to be similar to the original maintaining the resistance of the willow. The authors suggest that the willow rapidly exerts strong selective pressures in the rhizosphere, selecting for a similar microbiome from starting microbiomes. Recently, Chaudhary et al. (2019) showed that indigenous soil microbial community structure was not disturbed by the external application of exogenous microorganisms, despite their were able to improve plant growth promoting traits (i.e., nutrients availability, phosphatase activity). Therefore, the incorporation of exogenous microbiomes or beneficiary microorganisms does not perturb the natural soil microbial community.

## Concluding Remarks and Future Prospective

The new metaomics techniques have significantly increased our knowledge about the dynamics and abundance of microbial communities in soil-plant systems. However, the complex interactions between microorganisms-plants-soil is largely unknown. In the current scenario of climate change, a new green revolution is required to achieve future food security, with new concepts and approaches to achieve a more sustainable development of agriculture. In this context, the study and use of complete microbiomes or core microbiomes from extreme environments could represent a promising alternative to increase crop yields under different stress conditions. These microorganisms could transfer their innate resistance to agricultural crops. Furthermore, news formulations of bioinoculants composed of microbiomes or core microbiomes could eliminate the ecological barriers imposed by native microbial communities in soil, increasing the persistence of added microorganisms. In addition, the formulation of bioinoculants from the natural selection imposed by plants, through the phenomenon of cry for help, where the plant in stress situations selects its microbiome represents an eco-friendly alternative for a new generation of bioinoculants taking advantage of the closed relationship between the microbiome and their hosts, which could represent an excellent alternative to improve the plant fitness to counteract the climate change effects.

## Author Contributions

RR wrote the main manuscript text. PD critically revised the manuscript and approved the final version.

## Conflict of Interest

The authors declare that the research was conducted in the absence of any commercial or financial relationships that could be construed as a potential conflict of interest.
